# Biomarkers expression among paired serous ovarian cancer primary lesions and their peritoneal cavity metastases in treatment‐naïve patients: A single‐center study

**DOI:** 10.1002/cam4.4600

**Published:** 2022-02-25

**Authors:** Ilias P. Nikas, Cheol Lee, Min Ji Song, Bohyun Kim, Han Suk Ryu

**Affiliations:** ^1^ School of Medicine, European University Cyprus Nicosia Cyprus; ^2^ Department of Pathology, Seoul National University Hospital Seoul Republic of Korea; ^3^ Center for Medical Innovation, Biomedical Research Institute, Seoul National University Hospital Seoul Republic of Korea; ^4^ Department of Pathology, Seoul National University College of Medicine Seoul Republic of Korea

**Keywords:** ascitic fluid, cytopathological techniques, extracellular matrix metalloproteinase inducer (EMMPRIN), immune checkpoint inhibitors, immunohistochemistry, neoplasm metastasis, prognosis

## Abstract

**Background:**

High‐grade serous ovarian carcinoma (HGSOC), the most common histologic subtype of ovarian epithelial cancer, is associated with treatment resistance, enhanced recurrence rates, and poor prognosis. HGSOCs often metastasize to the peritoneal cavity, while fluid cytology examination could identify such metastases. This retrospective study aimed to identify potential biomarker discrepancies between paired HGSOC primary tissues and metastatic peritoneal fluid cytology samples, processed as cell blocks (CBs).

**Methods:**

Twenty‐four pairs of formalin‐fixed, paraffin‐embedded primary tissues and metastatic CBs from an equal number of treatment‐naïve patients were used, and immunohistochemistry (IHC) for epidermal growth factor receptor (EGFR), human epidermal growth factor receptor, programmed cell death‐1 ligand 1 (PD‐L1), and CD147 was applied.

**Results:**

13/24 pairs showed discordant EGFR IHC results; in all these 13 patients, EGFR was positive (≥1+ membranous staining intensity found in at least 10% of the cancer cells) in the peritoneal, yet negative in the primary tissue samples. Notably, EGFR IHC was positive in 15/24 of the metastatic, whereas in just 2/24 of the primary HGSOC samples (p < 0.001). Although most PD‐L1 results were concordant, 5/24 and 6/24 pairs exhibited discordant results when stained with the E1L3N and 22C3 clones, respectively. Lastly, CD147 overexpression was found more often in the metastatic rather than the matched primary HGSOCs stained with CD147, though the difference was not significant.

**Conclusions:**

Cytology from effusions could be considered for biomarker testing when present, even when tissue from the primary cancer is also available and adequately cellular, as it could provide additional information of potential clinical significance.

## Graphical abstract

This study aimed to identify potential biomarker discrepancies between primary tissues with high‐grade serous ovarian cancer (HGSOC) and their paired peritoneal fluid metastases, processed as cell blocks. EGFR, HER2, PD‐L1, and CD147 immunohistochemical expression was compared between 24 HGSOC pairs. EGFR overexpression was found more commonly in the metastatic than the primary samples, at a significant level (*p* < 0.001). Cytology from effusions could be considered for biomarker testing when present, even when tissue from the primary cancer is also available and adequately cellular, as it could provide additional information of potential clinical significance.

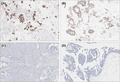

## 1 INTRODUCTION

Ovarian cancer (OC) is the leading cause of death among tumors of the gynecologic tract in the US and worldwide.^1^, ^2^ Most cases are diagnosed in an advanced stage, often due to the absence of early symptoms and detection methods, while they are managed with debulking surgery followed by platinum‐based chemotherapy. However, recurrence and metastasis rates are high because of treatment resistance—even if most patients initially respond—resulting in poor 5‐year survival rates.^3^ OCs exhibiting germline or somatic BRCA1 or BRCA2 mutations could be treated with Poly (ADP‐ribose) polymerase inhibitors, while other targeted therapies such as anti‐angiogenic agents (e.g., bevacizumab) and immune checkpoint inhibitors (ICIs) have shown promise.^3^, ^4^ The most common and lethal OC histologic subtype is the high‐grade serous ovarian carcinoma (HGSOC), which presents in older women and is characterized by the presence of p53 mutations.^1^, ^2^, ^5^ Immunohistochemistry (IHC) is often used in diagnostic routine practice to subclassify the diverse OC subtypes or distinguish them from metastases to the ovaries.^5^ During cancer progression, HGSOC often disseminates into the peritoneal cavity, forming metastases.^6^ In this case, patients could present with ascites, while the cytologic examination of peritoneal fluid could identify metastatic malignant cells.^7^ Pathology laboratories apply diverse techniques to process peritoneal fluids and enhance their diagnostic accuracy. With cell block (CB) preparations, pathology laboratories can fix peritoneal fluid samples with formalin and subsequently embed them in paraffin blocks.^7^, ^8^ There are several techniques to prepare CBs, which generally allow the already standardized histology protocols to be used in cytologic material to evaluate morphology (with hematoxylin and eosin staining) or apply IHC and other molecular techniques.^7^, ^8^, ^9^

Epidermal growth factor receptor (EGFR) and human epidermal growth factor receptor 2 (HER2) belong to the ErbB family of receptor tyrosine kinases, which are involved in key processes regulating cellular growth, differentiation, and survival.^10^ EGFR alterations in cancer include mutations—for instance in non‐small cell lung cancer (NSCLC)—managed with tyrosine kinase inhibitors (TKIs), or overexpression (e.g., in colorectal cancer), often managed with monoclonal antibodies (mAbs). EGFR wild‐type overexpression has been linked to disease progression and dismal prognosis in various cancers.^11^ Similarly, HER2‐positive cancers can also be targeted with mAbs (e.g., trastuzumab) or TKIs (e.g., lapatinib), respectively.^12^ The programmed cell death protein 1 (PD‐1) and its ligand, PD‐1 ligand 1 (PD‐L1), comprise a principal immune checkpoint pathway that regulates immune homeostasis in health and disease. PD‐L1 binding to PD‐1 suppresses the T‐cell response, leading to immune tolerance. However, blockage of the PD‐1/PD‐L1 interaction by ICIs could restore the function of exhausted T‐cells, re‐establish their anticancer activity, and prolong survival.^13^, ^14^ CD147, or EMMPRIN (extracellular matrix metalloproteinase inducer), is a human immunoglobulin that enhances the production of matrix metalloproteinases (MMPs), regulating the tumor microenvironment (TME). It is overexpressed in diverse malignancies and regarded as a prognostic biomarker and potential therapeutic target.^15^, ^16^

A few published studies have shown biomarker discrepancies among paired primary and metastatic lesions that could have significant clinical implications, affecting prognosis, or modifying treatment strategies.^17^, ^18^, ^19^ Such heterogeneity between primary and metastatic lesions could drive tumor progression and therapy resistance.^20^ To our knowledge, a comparison of EGFR IHC expression between primary OCs and their metastases has been reported by one study.^21^ In addition, although therapies with single‐agent EGFR inhibitors have not shown success in OC,^22^ combination with other modalities are being investigated in clinical trials (e.g., NCT04429542). HER2 overexpression has been associated with poor survival in OC patients,^23^ while its expression between matched primary lesions and metastases has been reported by one group.^24^ Similarly, only a few studies have correlated the PD‐L1 immunohistochemical status between primary OC lesions and their paired metastases.^25^, ^26^, ^27^ Lastly, CD147 overexpression has been reported in diverse malignancies and linked with dismal prognosis and resistance to chemotherapy,^15^, ^28^ a common issue of HGSOC management.^3^ This study aimed to compare, also identify potential EGFR, HER2, PD‐L1, and CD147 discrepant IHC results between paired HGSOC primary tissues and metastatic peritoneal fluid samples processed as CBs.

## 2 MATERIALS AND METHODS

The electronic medical records of the Seoul University Hospital (SNUH) Pathology Department were searched for paired HGSOC primary cancers and peritoneal metastases diagnosed with CB cytology from 2011 to 2020. All patients were treatment‐naive at the time of primary ovarian tissue or peritoneal fluid collection. The following clinicopathological parameters were collected: Age, FIGO stage at diagnosis, type of primary therapy, any additional targeted therapy, response after primary therapy (complete response; partial response; progressive disease), progression‐free survival (PFS) in months, and CA‐125 levels at diagnosis and after primary therapy.

Formalin‐fixed, paraffin‐embedded (FFPE) histology and cytology blocks from the abovementioned patients were collected from the archive, while 4 μm thick sections were cut from each sample to be used for subsequent IHC staining. Cytology CBs had been prepared during routine practice using a pre‐gelatinized starch technique.^29^ Before processing, all cases were assessed for tumor cellularity. To be included in the study, each histology and CB case had to contain at least 100 cancer cells.^30^ The following IHC stains were applied on whole slides from both primary and metastatic lesions of each patient, using an automated BenchMark ULTRA System (Roche Diagnostics) and the same protocol in both histology and cytology blocks: EGFR (790–2988; Ventana; RTU), HER2 (790–4493; Ventana; RTU), CD147 (GTX20666; GeneTex; 1:75), PD‐L1 (E1L3N; Cell Signaling; 1:100), and PD‐L1 (22C3; Concentrate; Dako; 1:50). All slides were counter‐stained with Harris hematoxylin. EGFR was considered positive (overexpressed) if a ≥1+ membranous staining intensity was found in at least 10% of the OC cells.^31^ HER2 was scored according to the latest American Society of Clinical Oncology‐College of American Pathologists (ASCO‐CAP) guidelines as 0, 1+, 2+, and 3+.^32^ A semi‐quantitative “H‐score” was used to evaluate the CD147 membranous immunostaining, taking into account both staining intensity and percentage of cells at each intensity level (formula: [1 × (% cells 1+) + 2 × (% cells 2+) + 3 × (% cells 3+)]), and ranging from 0 to 300.^33^, ^34^ PD‐L1 scoring was performed using the Tumor Proportion Score (TPS) for both E1L3N and 22C3 antibodies and classified as TPS < 1% and TPS > 1%, respectively.^35^ All slides were scanned with the Aperio Digital Pathology Slide Scanner AT2 (Leica Biosystems), while the QuPath platform for bioimage analysis was used to evaluate all immunostains.^36^ The IHC expression of EGFR, HER2, CD147, and PD‐L1 was compared between the primary and their paired metastases with Fisher's exact test. The MedCalc statistical software for Windows version 19.4 (MedCalc Software, Ostend, Belgium) was used. This study received approval from the SNUH Institutional Review Board; the committee also waived the requirement to obtain informed consent from the patients.

## 3 RESULTS

### 3.1 Patient characteristics

Forty‐eight HGSOC blocks from paired primary (*n* = 24) and peritoneal metastatic lesions (*n* = 24), diagnosed in the SNUH Pathology Department, were used in this study (Table 1). The mean age of the 24 study participants was 55.17 years. Sixteen patients were diagnosed at FIGO stage IIIC, while five of them were at stage IV. CA‐125 levels ranged from 61.2 to 11,630 U/ml at diagnosis, whereas from 2.7 to 753 U/ml after primary therapy. Seventeen patients showed complete response, while five of them had partial response and two had progressive disease after primary therapy. Despite therapy, 21/24 patients recurred during follow‐up and the PFS ranged from 3 to 38 months (mean: 21.33 months).

**TABLE 1:** Clinicopathological characteristics of the 24 patients included in the study

Patient	Age at Dx	FIGO stage at Dx	Response after primary therapy	CA‐125 levels at Dx (U/ml)	CA‐125 levels after primary therapy (U/ml)	PFS (months)
1	61	IIIC	CR	61.2	5.7	38
2	62	IIIC	CR	276.5	8.3	20
3	58	IIC	CR	1007	2.7	15
4	53	IIIC	PR	319	19	27
5	42	IIIC	PR	3895	55.8	15
6	48	IV	CR	330.5	6.4	31
7	60	IIIC	CR	162.4	8	13
8	58	IIIC	PD	2416	36	15
9	45	IIIB	CR	488.2	9.2	10
10	75	IIIC	PR	193.8	10.6	10
11	42	IIIC	PR	352	18.6	21
12	52	IIIC	CR	559.56	6	18
13	54	IIIC	CR	1881.2	8	26
14	55	IV	CR	6900	14.3	11
15	72	IIIC	CR	310.6	3.7	25
16	48	IVB	CR	3780	3.9	17
17	71	IIIC	PR	1593	11.4	19
18	42	IIIC	PD	2989	753	3
19	42	IIIC	CR	655.8	5.9	29
20	63	IIIB	CR	1465	6.9	27
21	56	IVB	CR	11,630	5.1	21
22	48	IVB	CR	81	2.7	36
23	61	IIIC	CR	213	4.9	37
24	56	IIIC	CR	526	2.8	28

Abbreviations: CR, complete response; PD, progressive disease; PFS, progression‐free survival; PR, partial response.

### 3.2 Epidermal growth factor receptor

**FIGURE 1:**
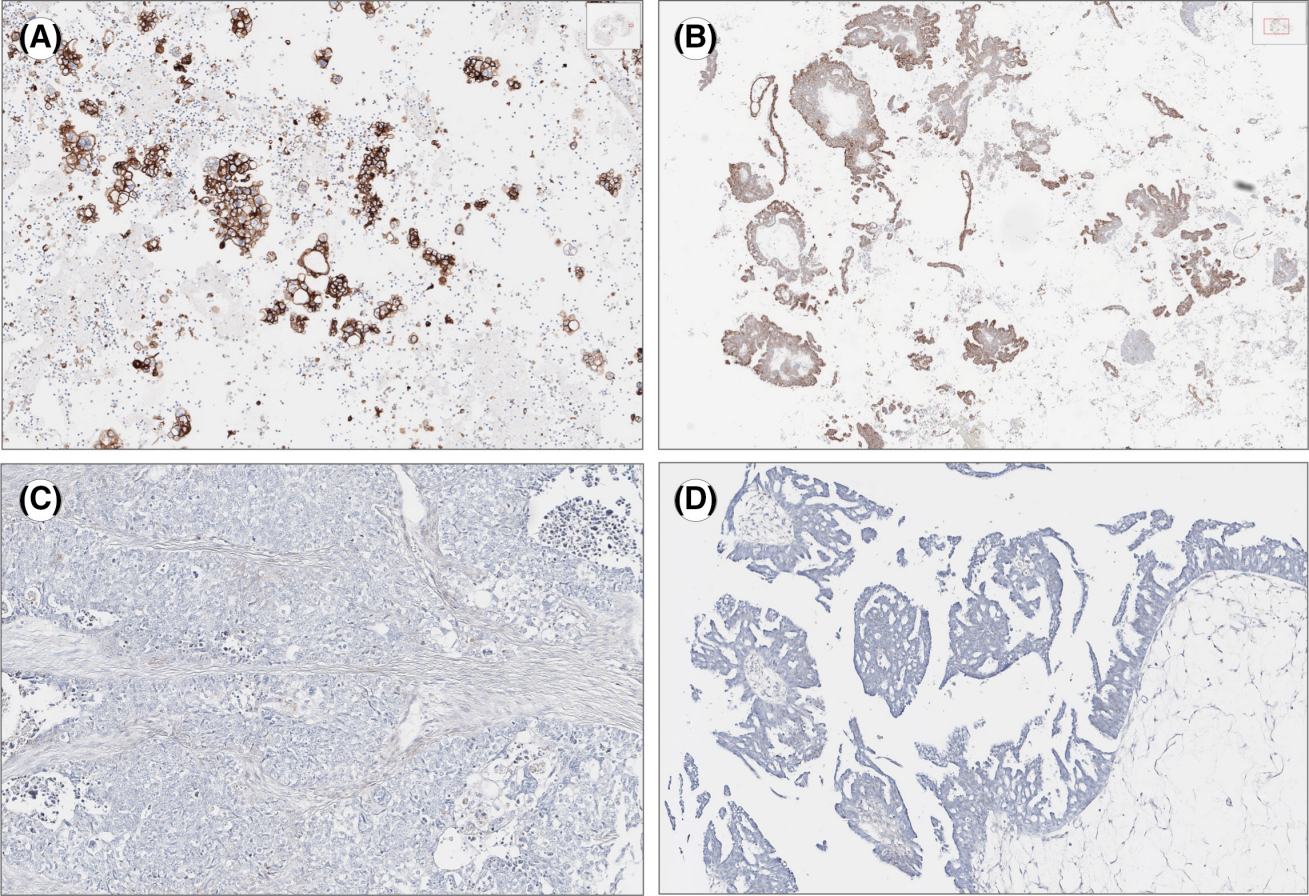
Two high‐grade serous ovarian cancer metastasis samples, processed as cell blocks, showed higher EGFR expression (A and B; immunohistochemistry) compared to their paired primaries (C and D; immunohistochemistry). These cases were evaluated and photographed using the QuPath platform for bioimage analysis (https://qupath.github.io/)

EGFR IHC results were largely discordant between the paired primary and metastatic HGSOC samples (Table 2; 13/24; 54%). Notably, in all these 13 patients, EGFR was overexpressed in the peritoneal, whereas it was negative in their matched primaries (Figure 1). EGFR was positive in 15/24 of the metastatic, albeit in just 2/24 primary lesions (*p* < 0.001). Eleven out of 24 pairs showed concordant EGFR IHC results; of them, 2/24 exhibited overexpression.

**TABLE 2:** Immunohistochemistry (IHC) results among the paired primary (P) and metastatic (M) high‐grade serous ovarian cancers from each of the 24 patients included in the study

Patient	EGFR (P)	EGFR (M)	CD147 (P)	CD147 (M)	PD‐L1 (P) E1L3N	PD‐L1 (M) E1L3N	PD‐L1 (P) 22C3	PD‐L1 (M) 22C3
1	neg	neg	2.5	3	<1%	>1%	<1%	>1%
2	neg	pos	3	3	<1%	<1%	<1%	<1%
3	neg	pos	3	3	>1%	<1%	>1%	<1%
4	neg	pos	2.7	3	<1%	<1%	<1%	<1%
5	neg	pos	2	2	<1%	<1%	<1%	<1%
6	neg	neg	3	3	<1%	<1%	<1%	<1%
7	neg	pos	2	3	<1%	<1%	<1%	<1%
8	neg	neg	3	3	>1%	<1%	>1%	<1%
9	pos	pos	3	3	<1%	<1%	<1%	>1%
10	neg	neg	3	3	<1%	<1%	<1%	<1%
11	neg	pos	3	3	<1%	<1%	<1%	<1%
12	neg	neg	1	2	<1%	<1%	<1%	<1%
13	neg	neg	3	3	<1%	<1%	<1%	<1%
14	neg	neg	0.5	3	<1%	<1%	<1%	<1%
15	neg	pos	3	3	<1%	<1%	<1%	<1%
16	neg	pos	2.5	2.5	<1%	>1%	<1%	>1%
17	neg	pos	3	3	<1%	<1%	<1%	<1%
18	neg	neg	0	0	<1%	<1%	<1%	<1%
19	neg	pos	1.5	1.5	<1%	<1%	<1%	<1%
20	neg	neg	3	3	<1%	<1%	<1%	<1%
21	pos	pos	2	1	>1%	<1%	>1%	<1%
22	neg	pos	3	3	<1%	<1%	<1%	<1%
23	neg	pos	0	0	<1%	<1%	<1%	<1%
24	neg	pos	3	3	<1%	<1%	<1%	<1%

*Note*: The metastatic cancers were processed as peritoneal fluid cell blocks. EGFR was considered positive if a ≥1+ membranous staining intensity was found in at least 10% of the ovarian cancer cells. A semi‐quantitative “H‐score” was used to evaluate CD147 IHC, considering both staining intensity and percentage of cells at each intensity level (formula: [1 × (% cells 1+) + 2 × (% cells 2+) + 3 × (% cells 3+)]), and ranging from 0 to 300. PD‐L1 scoring was performed using the Tumor Proportion Score (TPS) for both E1L3N and 22C3 antibodies and classified as TPS < 1% and TPS > 1%, respectively. HER2 IHC was also performed and scored according to the latest American Society of Clinical Oncology‐College of American Pathologists (ASCO‐CAP) guidelines as 0, 1+, 2+, and 3+, yet no discordant cases were identified; 23 pairs had a 0/1+ and one pair (patient 21) 2+ staining intensity.

Abbreviations: neg, negative; pos, positive.

### 3.3 Human epidermal growth factor receptor 2

**FIGURE 2:**
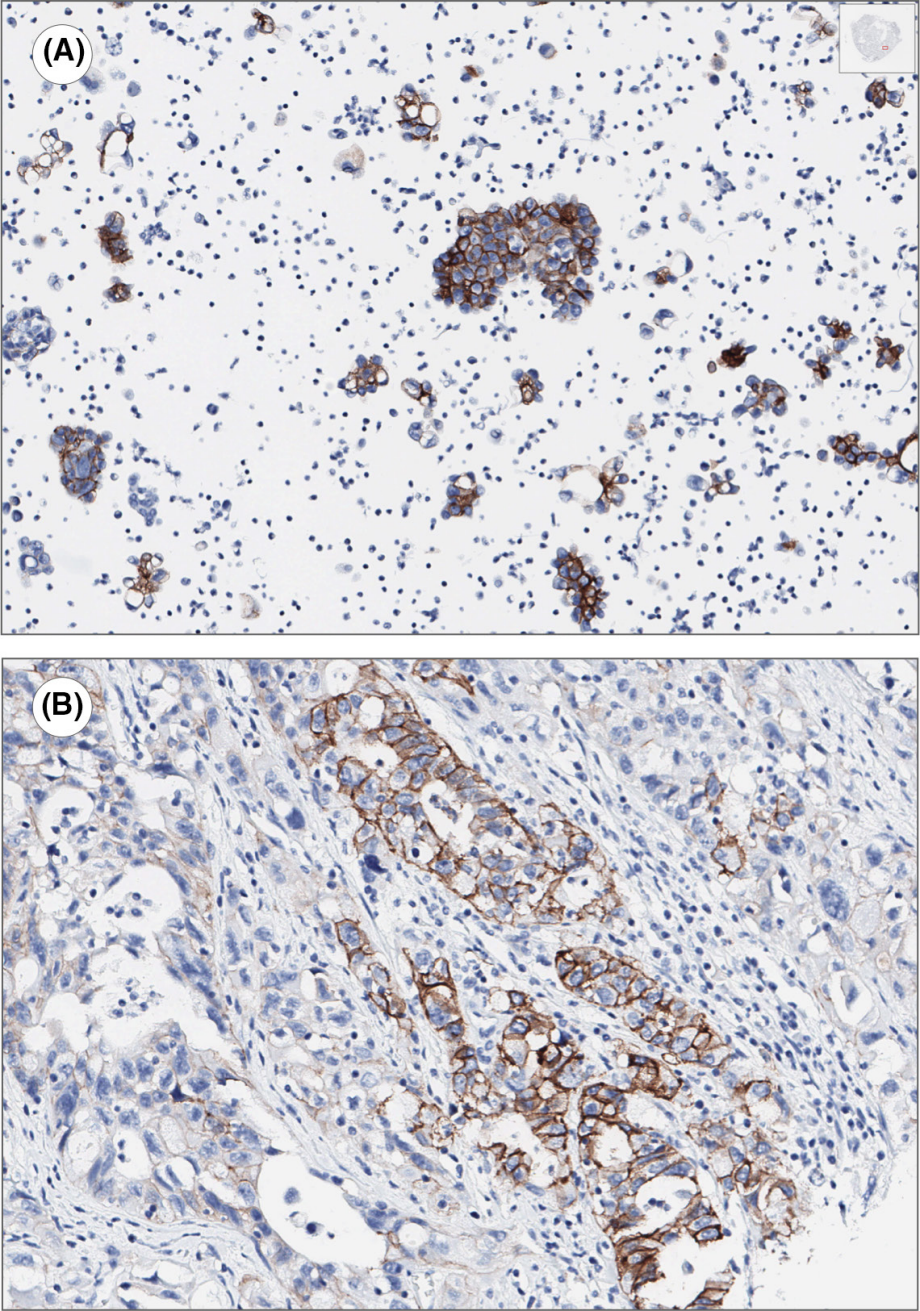
A high‐grade serous ovarian cancer metastasis sample, processed as cell block, showed a similar 2+ HER2 expression (A; immunohistochemistry) compared to its paired primary (B; immunohistochemistry). This case was evaluated and photographed using the QuPath platform for bioimage analysis (https://qupath.github.io/)

All paired cases showed concordant HER2 IHC results (24/24, 100%); 23/24 displayed low 0/1+ expression, while a 2+ HER2 expression was found in both ovarian and peritoneal effusion samples of a patient (Figure 2).

### 3.4 Programmed cell death‐1 ligand 1

**FIGURE 3:**
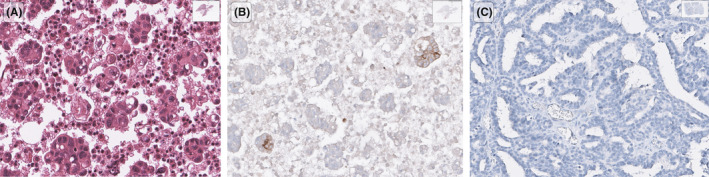
A high‐grade serous ovarian cancer metastasis sample, processed as cell block (A; H&E), showed a higher PD‐L1 expression (B; immunohistochemistry; E1L3N clone) compared to its paired primary (C; immunohistochemistry). This case was evaluated and photographed using the QuPath platform for bioimage analysis (https://qupath.github.io/)

Two PD‐L1 antibodies were used to assess the TPS in the paired HGSOC samples (Table 2). With the E1L3N clone, IHC results were concordant in 19/24 pairs (79%). For the rest five pairs, three were positive (TPS score > 1%) in the primary and negative (TPS score < 1%) in the metastatic lesions, while two pairs showed a TPS score > 1% only in the metastatic lesion (Figure 3). With the 22C3 clone, results were concordant in 18/24 pairs (75%). For the rest six pairs that discordance was detected, three were positive only in their primary (TPS score > 1%) and three only in their metastatic lesions. Most pairs exhibited a TPS score < 1% when stained with both antibodies, E1L3N (21/24 cases in the primary and 22/24 in the metastatic lesions) and 22C3 (21/24 cases in both primary and metastatic lesions). No sample showed a TPS score > 50%.

### 3.5 CD147

**FIGURE 4:**
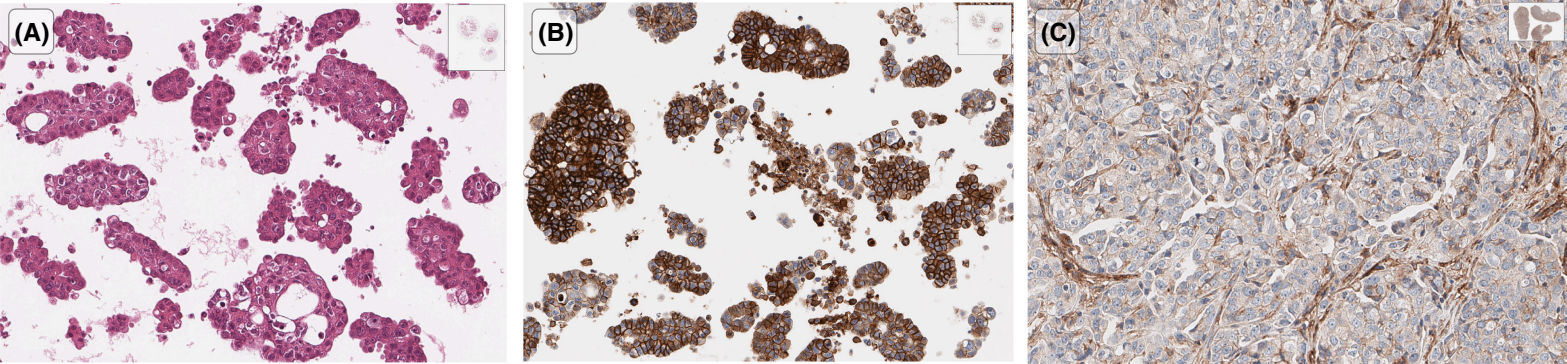
A high‐grade serous ovarian cancer metastasis sample, processed as cell block (A; H&E), showed a higher CD147 expression (B; immunohistochemistry) compared to its paired primary (C; immunohistochemistry). This case was evaluated and photographed using the QuPath platform for bioimage analysis (https://qupath.github.io/)

Results were concordant in 18/24 pairs (Table 2). Most cases exhibited CD147 overexpression, while the highest score (*H*‐score = 3) was found more often in the metastatic (Figure 4), rather than their matched primary lesions (17/24 vs. 13/24, respectively; *p* = 0.25).

## 4 DISCUSSION

Despite its small sample size, this study showed some discordant IHC results among the matched primary and peritoneal samples tested. Notably, EGFR IHC was largely discordant between the paired primary and metastatic HGSOCs tested, while in all 13 patients with discordant results, EGFR was overexpressed only in the peritoneal CB samples. In addition, EGFR IHC was overexpressed in 15/24 of the metastatic, whereas in just 2/24 of the primary samples, a statistically significant difference (*p* < 0.001). Although most PD‐L1 results were concordant, 5/24 and 6/24 pairs exhibited discordant results when stained with the E1L3N or 22C3 clones, respectively. Lastly, an *H*‐score = 3 was found more often in the metastatic rather than their matched primary HGSOCs stained with CD147 (17/24 vs. 13/24, respectively; *p* = 0.25). Cytology, especially when it utilizes CBs and has adequate cellularity, could be considered for biomarker testing either together or separately (e.g., when a tissue biopsy is difficult to obtain or unsuitable for molecular testing), as it could provide additional information of clinical importance.^37^, ^38^

Biomarker discrepancies in our study could have been due to technical reasons.^39^ Although we used only FFPE blocks—derived from HGSOC primary histology samples and peritoneal metastases processed as CBs—and identical IHC protocols to minimize technical variations and make our findings comparable, specimen handling could still differ in any step of this study or during the previous routine diagnostic preparation (e.g., duration of fixation), potentially impacting our results. Although cytology generally provides excellent material for IHC and molecular testing,^8^, ^40^ CB cytology samples in this study showed lower tumor cellularity than their paired histology, albeit both exhibiting adequate numbers of cancer cells for analysis (at least 100 cancer cells in all examined cases were needed as an inclusion criterion). This could have affected the subsequent IHC interpretation.^39^

Despite potential technical reasons though, such observed biomarker discrepancies could depict tumor heterogeneity between primary and metastatic lesions, a frequent phenomenon linked with tumor progression and therapy resistance.^20^, ^41^, ^42^ Several groups have reported discrepancies when comparing biomarker expression between paired primary and metastatic tissues in various cancers, for instance HER2 discrepancies in breast,^18^ gastric,^17^ endometrial,^43^ and colorectal cancers.^44^ These changes could have vital clinical implications, affecting prognosis or modifying treatment strategies.^17^, ^18^ In breast cancer, such biomarker conversions in the metastatic deposits could be associated with a more aggressive phenotype than their primary lesions. Similar discrepancies have also been reported for EGFR^39^, ^45^ or PD‐L1.^46^

In OC, research has shown that matched primary and metastatic OCs might show heterogeneous mutational and phenotypic profiles—even when both are sampled at the same time while the patients are in treatment‐naïve status—as a result of tumor evolution.^19^, ^47^ Metastatic OC deposits have shown genetic changes reflecting a more aggressive nature than their paired primary lesions; thus, they could indicate the current disease biology more precisely and offer additional information to oncologists when tested to assess prognosis and select targeted therapy or immunotherapy.^19^, ^25^

Our study revealed higher EGFR expression in a most metastatic HGSOC cases compared with their matched primary cancer deposits, which could have potential clinical significance. To our knowledge, one study has correlated the EGFR expression between primary OCs and their paired metastases; authors used a 1% membranous cut‐off (in contrast to the 10% we used) and found EGFR positivity in 28% and 33% of the primary and metastatic OC lesions, respectively.^21^ EGFR overexpression in OC has been associated with high tumor grade and poor prognosis in some studies, yet the response to EGFR targeted therapies seems limited.^48^, ^49^, ^50^ In contrast, other studies have not linked it with survival.^50^, ^51^ Of interest, although therapies with single‐agent EGFR inhibitors have yet been unsuccessful in OC management,^22^ combinations with other modalities is being studied in clinical trials (e.g., NCT04429542).

HER2 overexpression in OC seems to be an uncommon phenomenon. Similar to our study, where we noticed a low (0/1+) HER2 IHC expression in 23/24 pairs, Tuefferd et al. found HER2 overexpression in just 6.6% of the cases tested. In addition, they reported no significant difference in the HER2 expression between primary lesions and metastases.^24^ Notably, a recent meta‐analysis has shown that HER2 overexpression is associated with poor OS and DFS in OC patients.^23^

Accumulating evidence suggests that, in OC, PD‐L1 is mostly expressed in tumor‐associated macrophages (TAMs), while IHC labeling of tumor cells is relatively uncommon.^27^, ^52^ Our study agrees with Eymerit‐Morin et al., who reported that PD‐L1 tumor cell labeling in OC is an uncommon event and a TPS score > 1%, using the E1L3N antibody, was found in just 14% of their cases.^35^ Webb et al. also reported PD‐L1 tumor cell positivity in just 13.2% of the tested OC cases, while TAMs were labeled in most of their cases.^52^ Three recent studies correlated PD‐L1 immunohistochemical status between primary OC lesions and their paired metastases (Table 3); all of them utilized tissue rather than cytologic samples. Similar to our study, Gottlieb et al. reported PD‐L1 tumor cell IHC positivity in just 8% of their cases, whereas most exhibited positivity in the TAMs. While contrasting PD‐L1 tumor cell expression in primary versus metastatic sites, 13/16 treatment‐naïve pairs were concordant; the other three only exhibited positivity in the metastatic site.^27^ Bekos et al. showed that PD‐L1 IHC expression in the tumor cells of primary OC lesions was associated with the expression in metastatic peritoneal lesions (Spearman's coefficient = 0.540; *p* < 0.001), while its overexpression in TILs with a shorter overall survival (OS).^25^ Of interest, Parvathareddy et al. used a combined rather than a tumor cell PD‐L1 score and reported a positive PD‐L1 IHC expression in 44/125 (35.2%) HGSOC primary, albeit in 65/125 (52%) of the metastatic lesions tested.^26^ Conflicting results have been published concerning the prognostic role of PD‐L1 overexpression in OC, with studies linking it with poor^53^, ^54^, ^55^ or favorable prognosis.^52^, ^56^, ^57^ Notably, Huang et al. reported in a meta‐analysis that PD‐L1 overexpression was a poor prognostic factor in Asian countries, whereas a favorable in OC patients from non‐Asian countries.^58^

**TABLE 3:** Recent studies correlating PD‐L1 immunohistochemical status between primary ovarian cancer lesions and their paired metastases

Author, year	No. of cases	Type of samples	PD‐L1 clone	Relevant Findings
Bekos, 2021	111 (63 HGSOCs); 53 of them were paired OC primaries and peritoneal metastases	tissue biopsies (TMAs)	E1L3N	The PD‐L1 IHC expression in the tumor cells of primary OC lesions was associated with PD‐L1 expression in metastatic peritoneal lesions (Spearman's coefficient = 0.540; *p* < 0.001) High PD‐L1 IHC expression in TILs (>15% among TILs) was associated with poor OS (HR: 3.00 [1.05–8.72]; *p* = 0.041)
Parvathareddy, 2021	195 OC cases; 125 of them were HGSOC, paired OC primaries and peritoneal metastases	tissue biopsies (TMAs)	E1LN3; 1/100 dilution	Positive PD‐L1 IHC expression (≥5% positive cells in the cores) was found in 44/125 (35.2%) of the HGSOC primary, whereas in 65/125 (52%) of the metastatic lesions Positive PD‐L1 IHC expression in the primary OC lesions was associated with high stage (lymph node metastasis), whereas in the metastatic peritoneal lesions with high grade and ki‐67 status
Gottlieb, 2017	21 HGSOC cases, paired primaries and various metastases (e.g., omental, mesenteric, bowel); 16 of them were treatment‐naive	tissue biopsies (whole sections)	SP142; 1/200 dilution	PD‐L1 IHC expression was uncommon in tumor cells, whereas was often found in TAMs PD‐L1 was expressed (≥1%) in the tumor cells of 2/16 of treatment‐naive HGSOC primary and 5/16 of metastatic lesions (discordance in 3/16 cases)

Abbreviations: HGSOC, high‐grade serous ovarian cancer; IHC, immunohistochemistry; OC, ovarian cancer; OS, overall survival; TAMs, tumor associated macrophages; TILs, tumor infiltrated lymphocytes; TMAs, tumor microarrays.

In accordance with our study, Davidson et al. showed that CD147 overexpression at the mRNA level was more common in peritoneal metastatic lesions rather than primary ovarian tumors within their cohort.^59^ CD147 overexpression has been reported in diverse malignancies, including breast, lung, melanoma, urothelial, and OC. Notably, CD147 activates a few MMPs which degrade the TME, resulting in tumor progression, metastasis, angiogenesis, drug resistance, and dismal prognosis.^15^, ^28^, ^60^ A meta‐analysis has linked CD147 overexpression with reduced OS, DFS, and PFS, besides resistance to chemotherapy.^28^

This study has some important limitations. It was a single‐center retrospective study of small size, and all patients were of Asian descent; thus, our findings might not apply to other populations. In addition, although all primary ovarian and metastatic peritoneal samples used for this analysis were FFPE and the same IHC protocols were applied, technical reasons at any level and differences in the cellularity among the paired primary biopsy and metastatic cytology samples could still have impacted our results.

In conclusion, biomarker IHC testing performed on peritoneal fluid CBs exhibited some discrepancies when compared with the IHC results in their paired primary HGSOCs, a finding of potential clinical significance. Cytology CBs from peritoneal effusions could be considered for biomarker testing when present, even when a biopsy of the primary OC tissue is available. As our study showed, testing on the cytologic material could reveal new findings, such as the EGFR overexpression in some peritoneal HGSOC metastases. Future studies of prospective design, using larger patient cohorts, may provide more solid evidence for future clinical practice.

## CONFLICT OF INTEREST

The authors declare no competing financial interests.

## ETHICS STATEMENT

This study received approval from the SNUH Institutional Review Board; the committee also waived the requirement to obtain informed consent from the patients.

## Data Availability

The data that support the findings of this study are available from the corresponding author upon reasonable request.
